# Distribution of transferrin binding protein B gene (*tbpB*) variants among *Neisseria *species

**DOI:** 10.1186/1471-2180-8-66

**Published:** 2008-04-22

**Authors:** Odile B Harrison, Martin CJ Maiden, Bachra Rokbi

**Affiliations:** 1 The Peter Medawar Building for Pathogen Research and Department of Zoology, University of Oxford, South Parks Road, Oxford, OX1 3SY, UK; 2 Sanofipasteur, 69280 Marcy l'Etoile, France

## Abstract

**Background:**

Transferrin binding protein B (*tbpB*), an outer membrane lipoprotein, is required for the acquisition of iron from human transferrin. Two *tbpB *families have been documented in *Neisseria meningitidis*: an isotype I *tbpB *gene of 1.8 kb and an isotype II *tbpB *gene of 2.1 kb, the former expressed by meningococci in the disease-associated ST-11 clonal complex and the latter found among meningococci belonging to the hyper-invasive clonal complexes including ST-8, ST-18, ST-32, ST-41/44 as well as *N. gonorrhoeae *isolates. The origin of the isotype I *tbpB *gene is unknown, however several features in common with non-pathogenic *Neisseria *and the ST-11 clonal complex *N. meningitidis *isolate FAM18 have been documented leading to the hypothesis that the isotype I *tbpB *gene may also be shared between non-pathogenic *Neisseria *and ST-11 meningococci. As a result, the diversity of the *tbpB *gene was investigated in a defined collection of *Neisseria *species.

**Results:**

Two families of isotype I *tbpB *were identified: family A containing conserved genes belonging to ST-11 meningococci, *N. polysaccharea *and *N. lactamica *isolates and family B including more diverse isotype I *tbpB *genes from *N. sicca*, *N. mucosa*, *N. flava, N. subflava *as well as *N. cinerea, N. flavescens *and *N. polysaccharea *isolates. Three isotype II *tbpB *families were identified with: family C containing diverse *tbpB *genes belonging to *N. polysaccharea*, *N. lactamica, N. gonorrhoeae *and *N. meningitidis *isolates, family D including another subset of isotype II *tbpB *genes from *N. lactamica *isolates and family E solely composed of *N. gonorrhoeae tbpB *genes.

**Conclusion:**

This study reveals another instance of similarity between meningococci of the ST-11 clonal complex and non-pathogenic *Neisseria *with the origin of the isotype I *tbpB *gene resulting from a horizontal genetic transfer event occurring between these two populations.

## Background

The genus *Neisseria *contains 12 species and biovars colonising humans most of which are non-pathogenic colonisers of the upper respiratory tract [1,2]. Only two species, *Neisseria gonorrhoeae*, the etiological agent of gonorrhoea and *Neisseria meningitidis*, a major cause of meningitis and septicaemia worldwide, regularly cause disease in humans [3,4].

A major determinant in the survival of *Neisseria *within the human host is the ability to acquire iron, the majority of which is not circulating freely in the human body but is stored in ferritin and haemoglobin or is complexed with the glycoproteins transferrin and lactoferrin [5]. *Neisseria *have devised ways to counteract this iron limitation through the evolution of several high-affinity receptor systems including the lactoferrin binding proteins A and B, the transferrin binding proteins A and B, and the haptoglobin-haemoglobin receptor HpuAB, each composed of an accessory lipoprotein subunit and a TonB-dependent gated porin [6-11]. In addition, *Neisseria *can obtain iron through the expression of the surface-exposed haemoglobin receptor HmbR [12,13].

Based on antigenic and genomic features of TbpB and *tbpB*, *N. meningitidis *isolates can be classified into two major families: isotype I (*tbpB *gene of 1.8 kb and TbpB protein with a mass of approximately 68 kDa) and isotype II (*tbpB *gene of 2.1 kb and TbpB protein with a mass of approximately 80 to 90 kDa) [14]. Isotype II *tbpB *genes have been documented in several *N. meningitidis *clonal complexes including ST-8, ST-32, ST-18 and ST-41/44 as well as non-pathogenic *Neisseria *[14-17]. Isotype I *tbpB *genes, on the other hand, have solely been identified among *N. meningitidis *isolates belonging to the ST-11 clonal complex and have not been detected among other *Neisseria *species. Four *tbpB *families were recently described based on partial nucleotide sequences from serogroup A clonal complex ST-4 *N. meningitidis *and *N. lactamica *isolates collected in The Gambia [16,17]. Families one and four contained diverse isotype II *tbpB *alleles from *N. meningitidis *and *N. lactamica *isolates and families two and three included isotype I *tbpB *alleles. Importantly, among the 138 isolates analysed (98 serogroup A ST-4 meningococci, 12 unrelated meningococci, 22 *N. lactamica *isolates, and 6 unidentified *Neisseria *spp.) only three isotype I *tbpB *alleles were found, all of which belong to meningococci [16,17].

Meningococci from the ST-11 clonal complex have been a major cause of meningococcal disease worldwide throughout the last century and despite low carriage rates continue to be associated with disease [18]. In addition to the isotype I *tbpB *gene, ST-11 meningococci can be distinguished from other hyper-virulent clonal complexes by the absence of an *opcA *gene and the possession of a class 2 *porB *gene [19-21]. Furthermore, similarities between the ST-11 clonal complex isolate FAM18 and non-pathogenic *Neisseria *spp. have been reported including the clustering of *pilE *sequences [22] and the comparable genetic organisation of the *opcA *negative locus in two *N. polysaccharea *isolates [23]. Taken together, these observations indicate the occurrence of specific horizontal genetic exchange events between commensal *Neisseria *and ST-11 meningococci which may have contributed to the described genetic isolation of this clonal complex [24]. The origin of the isotype I *tbpB *gene is unknown. Consequently, the distribution of the gene in a defined collection of *Neisseria *spp. was investigated with the hypothesis that the isotype I *tbpB *gene was present in other *Neisseria *spp.

## Results

### Identification of the *tbpB *gene

Isotype I *tbpB *genes were isolated from the non-pathogenic *Neisseria *spp. Two families of the gene became apparent. The first contained sequences closely related to meningococcal ST-11 *tbpB *genes belonging to three *N. polysaccharea *and two *N. lactamica *isolates. The second included more divergent isotype I *tbpB *genes from the non-pathogenic *Neisseria *spp. *N. sicca*, *N. mucosa*, *N. flava*, *N. subflava*, *N. cinerea*, *N. flavescens *as well as from another three *N. polysaccharea *isolates (Table 1). Isotype II *tbpB *genes were obtained from the remaining six *N. lactamica *and two *N. polysaccharea *isolates, while in agreement with previous studies, *N. gonorrhoeae *isolates contained isotype II *tbpB *genes (Table 1) [25]. A further 23 non-pathogenic *Neisseria *isolates were analysed and found to be negative for the *tbpB *gene. Among these were *N. polysaccharea*, *N. perflava, N. sicca, N. subflava, N. flava *and *N. mucosa *isolates. These isolates may contain divergent or truncated *tbpB *genes unable to be amplified with the primers used, however the remainder of this study will focus on the *tbpB *genes that were sequenced.

**Table 1:** Summary of *tbpB *families and nomenclature used

**TbpB Family**	**size (kb)**	***tbpB* isotype**	**Previous designation [16, 17]**	***Neisseria *species**
*tbpB*_*A*_	1.8	I	Families 2 & 3	*N. meningitidis *clonal complex ST-11, *N. polysaccharea *and *N. lactamica*
*tbpB*_*B*_	1.8	I	ND	*N. polysaccharea*, *N. sicca*, *N. cinerea*, *N. mucosa*, *N. flava*, *N. subflava *and *N. flavescens*
*tbpB*_*C*_	2.1	II	Family 1	*N. meningitidis *belonging to the clonal complexes ST-32, ST-41/44, ST-8, ST-18, *N. lactamica*, *N. polysaccharea *and *N. gonorrhoeae*
*tbpB*_*D*_	2.1	II	Family 4	*N. meningitidis *and *N. lactamica*
*tbpB*_*E*_	2.1	II	ND	*N. gonorrhoeae*

Functional assessment of the protein was beyond the scope of the present study. Nevertheless, previously documented putative transferrin binding sites were observed based on predicted translations of the nucleotide sequences [26,27]. In particular, the highly conserved domains N3, N4 and C3, critical for efficient iron uptake and located in both the N- and C- terminal segments among isolates *N. gonorrhoeae *FA19, *N. meningitidis *M982, *Moraxella catarrhalis *4223 and *Acinetobacter pleuropneumoniae *serotype 7, were also identified [27]. Domain N3 (residues 377 to 388 in *N. gonorrhoeae *FA19) displayed 100% sequence identity in both isotype I TbpB families, whereas six non-synonymous changes were observed among isotype II TbpB. Domain N4 (residues 409 to 422 in *N. gonorrhoeae *FA19) was also highly conserved among isotype I TbpB with the occurrence of three non-synonymous substitutions. Five variable sites were present among isotype II TbpB. Domain C3 (residues 703 to 713 in *N. gonorrhoeae *FA19) showed the most diversity with the occurrence of four non-synonymous substitutions among both TbpB isotypes.

### Phylogenetic relationships inferred from novel *Neisseria tbpB *sequences

All of the sequences were aligned manually with sequences starting from and ending at the same amino acid residue in each isolate. Published isotype I and II *tbpB *sequences from isolates B16B6, M982, 8680, 8726, 2713, 2717 and FA19, used in previous analyses [14,16,17,27], were also included in the alignment as well as those from the sequenced genomes of *N. meningitidis *isolates FAM18, Z2491, MC58 and *N. gonorrhoeae *FA1090.

Phylogenetic analysis was undertaken using the software package ClonalFrame version 1.1, which is a statistical model-based method initially described for inferring bacterial clonal relationships using multilocus sequence data [28]. Inference is performed in a Bayesian framework and a neutral coalescent model is assumed based on the hypothesis that the bacteria in the sample come from a constant-sized population in which each bacterium is equally likely to reproduce, irrespective of its previous history. The key assumption of ClonalFrame is that recombination events introduce a constant rate of substitutions to a contiguous region of sequence with the end result that a clonal frame can be inferred. In the present study, over 50,000 iterations were performed with every hundredth tree sampled after which a 95% majority-rule consensus tree was derived. Annotation was then undertaken by importing the tree into the Molecular Evolutionary Genetics Analysis software package (MEGA version 4.0) [29].

**Figure 1:**
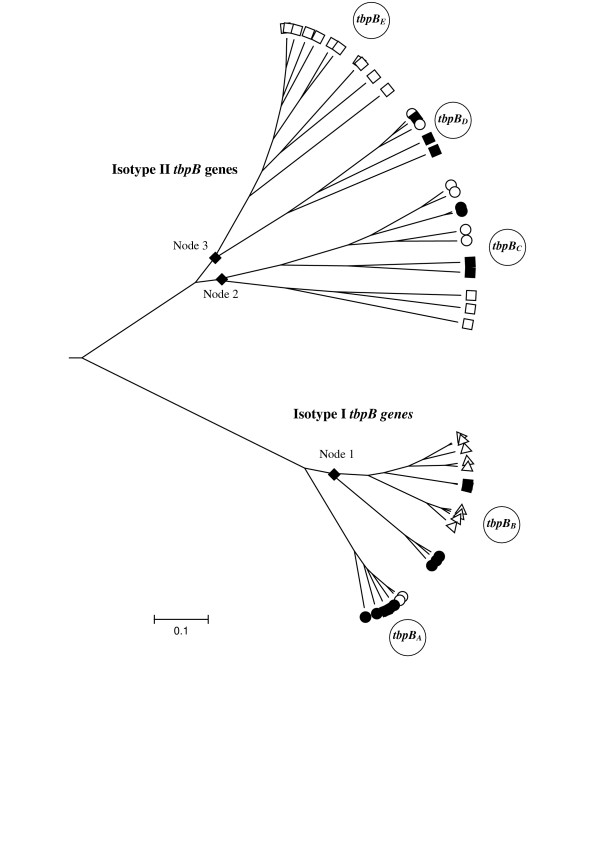
**Phylogenetic tree inferred from aligned *tbpB *genes**. Over 500 trees were generated using Clonalframe from which a 95% majority-rule consensus tree was derived and imported into MEGA version 4.0 for further annotation. Meningococcal reference *tbpB *genes (accession numbers in brackets) belonging to isolates B16B6, M982, 2713, 2717, 8710, 8680, FA19, FA1090, FAM18, Z2491 and MC58 [Genbank: Z15129, Z15130, AJ223044, AJ279554, Y09618, Y09977, U05205, U65219, AM421808, AL157959 and AE002098, respectively] were also included in the phylogenetic analysis. The proposed nomenclature for each *tbpB *family is indicated by large encircled letters. The nodes 1, 2 and 3 depicted with a diamond correspond to the recombination events presented in Figure 2. Open squares denote *N. gonorrhoeae tbpB *sequences, open circles *N. lactamica*, open triangles all of the other *Neisseria *spp. excluding *N. polysaccharea*, which are depicted with black circles and *N. meningitidis *which are represented by black squares.

The two major isotype families were evident with each family containing a distinct cluster of genes (Fig. 1). The shortness of the branches for isotype I *tbpB *genes indicated that these were a closely related group of sequences compared with the depth of the branches seen for isotype II *tbpB *genes where greater diversity is known to occur [30]. Closer inspection of the tree revealed the two families of isotype I *tbpB *genes observed by sequence analysis as well as three clusters of isotype II *tbpB *genes. For ease of interpretation, the two isotype I *tbpB *families have been named *tbpB*_*A *_and *tbpB*_*B *_with the isotype II clusters named *tbpB*_*C *_through to *tbpB*_*E *_(Fig. 1 and Table 1). This nomenclature is proposed according to published guidelines in bacterial genetics [31] and is recommended in light of the emergence of the new families revealed in this study. Hitherto, studies in *tbpB *genetic diversity have focussed on a specific *Neisseria *spp. or meningococcal clonal complex and have not encompassed all of the *Neisseria *species included in the present work. This inclusion has provided a more detailed analysis of *tbpB *diversity and will allow a more flexible nomenclature for *tbpB *genes.

Family *tbpB*_*A *_was comprised of genes most closely related to those of clonal complex ST-11 meningococci with four of these belonging to *N. lactamica *and *N. polysaccharea *isolates. Family *tbpB*_*B *_included a divergent cluster of isotype I genes (75% identity) belonging to a variety of *Neisseria *spp. as well as containing a subset of *N. polysaccharea *isolates (Fig. 1 and Tables 1 &2).

**Table 2:** *N. gonorrhoeae *and commensal *Neisseria *isolates used in this study

**Isolate**	**Site of isolation**	**Country of origin**	***tbpB *family**	**Genbank Accession No**	**Reference**
*N. gonorrhoeae *22584	genitourinary	USA	*tbpB*_*E*_	[AM849572]	This study
*N. gonorrhoeae *25562	DGI	unknown	*tbpB*_*E*_	[AM849573]	This study
*N. gonorrhoeae *26034	DGI	unknown	*tbpB*_*E*_	[AM849574]	This study
*N. gonorrhoeae *26399	DGI	unknown	*tbpB*_*E*_	[AM849575]	This study
*N. gonorrhoeae *26593	DGI	unknown	*tbpB*_*E*_	[AM849576]	This study
*N. gonorrhoeae *27806	DGI	UK	*tbpB*_*C*_	[AM849577]	This study
*N. gonorrhoeae *27886	genitourinary	Bangladesh	*tbpB*_*E*_	[AM849578]	This study
*N. gonorrhoeae *27921	genitourinary	Uzbekistan	*tbpB*_*E*_	[AM849579]	This study
*N. gonorrhoeae *28197	genitourinary	Russia	*tbpB*_*E*_	[AM849580]	This study
*N. gonorrhoeae *28622	genitourinary	UK	*tbpB*_*C*_	[AM849581]	This study
*N. gonorrhoeae *29528	genitourinary	UK	*tbpB*_*E*_	[AM849582]	This study
*N. gonorrhoeae *F62	genitourinary	USA	*tbpB*_*C*_	[AM849571]	This study
*N. gonorrhoeae *FA19	DGI	USA	*tbpB*_*E*_	[U05205]	[35]
*N. gonorrhoeae *FA1090	DGI	USA	*tbpB*_*E*_	[U65219]	[25]
*N. lactamica *8064	nasopharynx	France	*tbpB*_*C*_	[AM849588]	[40, 41]
*N. lactamica *2^nd^1	nasopharynx	Oman	*tbpB*_*D*_	[AJ704747]	This study
*N. lactamica *2^nd^94	nasopharynx	Oman	*tbpB*_*A*_	[AJ704737]	This study
*N. lactamica *2^nd^223	nasopharynx	Oman	*tbpB*_*D*_	[AM849585]	This study
*N. lactamica *2^nd^290	nasopharynx	Oman	*tbpB*_*C*_	[AJ704748]	This study
*N. lactamica *2^nd^291	nasopharynx	Oman	*tbpB*_*C*_	[AM849586]	This study
*N. lactamica *2^nd^292	nasopharynx	Oman	*tbpB*_*C*_	[AM849587]	This study
*N. lactamica *1^st^170	nasopharynx	Oman	*tbpB*_*A*_	[AJ704746]	This study
*N. flava *30008	nasopharynx	USA	*tbpB*_*B*_	[AJ704732]	This study
*N. subflava *9992	nasopharynx	USA	*tbpB*_*B*_	[AJ704745]	This study
*N. mucosa *ATCC 19696	sputum	unknown	*tbpB*_*B*_	[AJ704738]	[42, 43]
*N. sicca *ATCC 9913	unknown	unknown	*tbpB*_*B*_	[AJ704730]	[44]
*N. flavescens *ATCC 13120	CSF meningitis	USA	*tbpB*_*B*_	[AJ704733]	[45, 46]
*N. flavescens *414	unknown	France	*tbpB*_*B*_	[AJ704736]	[47]
*N. flavescens *ATCC 13119	CSF meningitis	USA	*tbpB*_*B*_	[AJ704734]	[48]
*N. flavescens *3536	CSF meningitis	USA	*tbpB*_*B*_	[AJ704735]	[48]
*N. cinerea *ATCC 14685	nasopharynx	Germany	*tbpB*_*B*_	[AJ704731]	[47, 49]
*N. polysaccharea *ATCC 43768	nasopharynx	France	*tbpB*_*B*_	[AJ704740]	[47, 49, 50]
*N. polysaccharea *90400	nasopharynx	Canada	*tbpB*_*B*_	[AJ704743]	[23, 51]
*N. polysaccharea *89356	nasopharynx	Canada	*tbpB*_*C*_	[AJ704762]	[52]
*N. polysaccharea *85322	nasopharynx	Germany	*tbpB*_*C*_	[AJ704761]	[23, 53]
*N. polysaccharea *87043	nasopharynx	Canada	*tbpB*_*A*_	[AJ704742]	[23, 44, 51, 52]
*N. polysaccharea *P4-A	nasopharynx	UK	*tbpB*_*B*_	[AJ704739]	[48]
*N. polysaccharea *P7-A	nasopharynx	UK	*tbpB*_*A*_	[AJ704741]	[48]
*N. polysaccharea *P8-A	nasopharynx	UK	*tbpB*_*A*_	[AJ704744]	[48]

Three distinct isotype II *tbpB *families were apparent (Fig 1 and Tables 1 &2). Several gonococcal genes have clustered together and can be found in family *tbpB*_*E *_with families *tbpB*_*C *_and *tbpB*_*D *_containing genes belonging to *N. lactamica, N. polysaccharea, N. gonorrhoeae *and *N. meningitidis *isolates. Throughout the tree isolates have clustered by *Neisseria *species indicative of within species conservation of *tbpB *genes. The Genbank accession numbers for new *tbpB *genes sequenced in this study are listed in Table 2 as well as those belonging to previously submitted *tbpB *sequences.

### Genetic diversity of the *tbpB *genes

Genes belonging to family *tbpB*_*A *_were the least diverse (mean *p-*distance ranging from 0.001 to 0.040) with 85 polymorphic sites, the majority of which occur among *N. polysaccharea *and *N. lactamica *isolates. Overall, six fixed differences were observed between the genes of ST-11 meningococci and those of *N. lactamica *and *N. polysaccharea *with no shared polymorphisms between the two populations. Family B *tbpB *genes were more diverse (mean *p-*distance value 0.117) with 415 polymorphic sites. In a comparison of both gene families, there were 210 fixed differences and 54 shared mutations.

As expected, families *tbpB*_*C*_, _*D *_and _*E *_were more diverse (mean *p-*distances = 0.187, 0.140 and 0.112 respectively). Genes belonging to the *N. polysaccharea *isolates shared 99% identity with 16 segregating sites, seven of which encoded non-synonymous changes. *N. lactamica tbpB *genes were more diverse with 696 polymorphic sites while 829 polymorphisms were observed among *N. gonorrhoeae tbpB *genes. A total of 445 shared mutations were observed between *N. lactamica *and *N. meningitidis tbpB*_*C *_genes indicative of recombination, while only six were apparent between *N. polysaccharea *and *N. meningitidis*.

**Figure 2:**
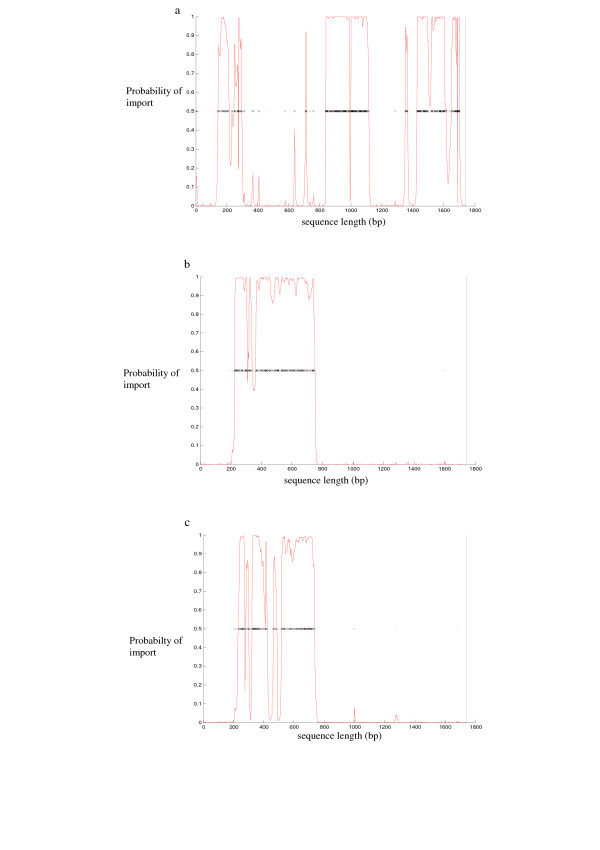
**ClonalFrame representation of *tbpB *recombination events**. The nucleotide sequence of *tbpB *genes is represented on the x axis with the red line indicating at each locus the probability for an import on a scale from 0 (bottom of the y axis) to 1 (top of the y axis). Each inferred substitution in the graph is represented by a cross, the intensity of which indicating the posterior probability for that substitution. In panel A, recombination events occurring at node 1 in the phylogenetic tree (fig. 1) are represented. A mosaic gene structure is evident with fragments present between bases 150 and 300, 800 and 1000 and 1400 and 1800. In panel B, horizontal genetic exchange at node 2 are depicted occurring from bases 200 to 800 and, in panel C node 3 is represented.

Very little recombination was noticeable among *tbpB*_*A *_genes or between these and family *tbpB*_*B*_, however a mosaic gene structure was present among the latter indicating that recombination occurred frequently among *tbpB*_*B *_genes from *N. sicca, N. flava, N. subflava, N. mucosa, N. flavescens, N. cinerea *and *N. polysaccharea *(Fig. 2a). As expected, *tbpB *genes from families *tbpB*_*C*, *D *_and _*E *_recombined often (Fig. 2b &2c) with most of this occurring from bases 200 to 800.

## Discussion

The aim of this study was to identify the origin of the isotype I *tbpB *gene. Previous observations have determined that these were confined to meningococci belonging to the ST-11 clonal complex [14,32]. In contrast, isotype II genes were widely distributed among *N. meningitidis *clonal complexes and *N. lactamica *isolates [14,16,17]. The results presented here reveal the existence of isotype I *tbpB *genes among diverse *Neisseria *spp. Based on phylogenetic analysis these could be divided into two families: *tbpB*_*A *_containing genes homologous to ST-11 meningococci and *tbpB*_*B *_including more distantly related isotype I genes belonging to diverse non-pathogenic *Neisseria *spp. (Table 1 and Fig. 1). *N. lactamica *and *N. polysaccharea *isolates were found with both *tbpB *isotypes while, in agreement with previous studies, *N. gonorrhoeae *isolates solely contained isotype II *tbpB *genes [25]. Phylogenetic analysis demonstrated the presence of three isotype II families, named *tbpB*_*C *_through to *tbpB*_*E*_. Family C contained genes belonging to *N. lactamica, N. polysaccharea, N. meningitidis *and *N. gonorrhoeae *isolates, family D included another subset of isotype II *tbpB *genes belonging to *N. lactamica *and *N. meningitidis *isolates and finally, family E comprised *N. gonorrhoeae *genes (Table 1 and Fig. 1). In light of the *tbpB *families now present a new nomenclature is proposed according to published guidelines in bacterial genetics [31]. Previously, studies in *tbpB *genetic diversity focussed on a specific *Neisseria *spp. or meningococcal clonal complex and did not encompass all of the *Neisseria *spp. included in the present work [14-17,25]. This inclusion has provided a more detailed analysis of *tbpB *diversity with the proposed nomenclature allowing more flexibility for future *tbpB *genes. Using this scheme genes can be grouped according to the family they belong to followed by an allele number.

A number of features are shared between clonal complex ST-11 *N. meningitidis *isolates and non-pathogenic *Neisseria*. Sequences upstream of the *pilE *gene from the class II pilin-producing *N. meningitidis *strain FAM18 were identical to the short region characterised upstream from *N. polysaccharea pilE *[22]. The *N. polysaccharea *isolate analysed (ATCC 43768) was included in the present study and harboured a *tbpB *gene similar to that of *N. meningitidis *isolate FAM18. Furthermore, o*pcA *genes are absent among meningococci belonging the ST-11 clonal complex and were also undetectable among *N. polysaccharea *isolates 87043 and 90400 [19,20], which were found in this study with isotype I *tbpB *genes. The identification of isotype I family A and B genes among *Neisseria *spp. is another characteristic shared with *N. meningitidis *isolates belonging to clonal complex ST-11 and is indicative of the occurrence of several horizontal genetic transfer events between non-pathogenic *Neisseria*, in particular *N. polysaccharea *and meningococci belonging to this clonal complex.

The evolutionary reasons leading to the existence of two *tbpB *isotypes among *Neisseria *are unknown. However, seclusion of isotype I *tbpB *to ST-11 clonal complex meningococci may be due to clonal expansion or selection for this isotype. Indeed, the isotype I TbpB protein has been shown to play an essential role in iron acquisition from human transferrin with isogenic mutants deficient in TbpB failing to grow on hTf as a sole iron source [33,34]. Thus, both the TbpA and TbpB parts of the transferrin complex are critical. This was reflected in the lower diversity observed among *tbpB *genes belonging to families A (mean *p-*distance ranging from 0.001 to 0.040) and B (mean *p-*distance 0.117), highlighting the importance of this gene in contributing to the fitness of the organism. There has been selection for isotype I *tbpB *among meningococci belonging to the ST-11 clonal complex such that it has become restricted to this clonal complex. In contrast, isotype II *tbpB *genes have been found to provide a purely facilitating role such that TbpB-deficient mutants were only incapacitated with slower growth [34]. This has been confirmed in isogenic mutagenesis studies of both TbpA and TbpB in *N. gonorrhoeae, H. influenzae *and *M. catarrhalis *(which all contain isotype II-like *tbpB *genes) [35-37]. The non-essential role the isotype II *tbpB *gene has in iron acquisition may contribute to its hyper-variability. Indeed, Zhu *et al *found that the high rate of import among isotype II *tbpB *genes, although providing a temporary advantage because of antigenic composition, resulted in reduced fitness of the isolates [16,17]. The higher recombination patterns observed in the present study among isotype II *tbpB *genes (Fig. 2b &2C) combined with the deeper phylogeny seen (Fig. 1) support this.

## Conclusion

This work investigated the distribution of the two *tbpB *variants among *Neisseria *spp. and aimed to discover the origin of the isotype I *tbpB *gene. Results revealed this gene was found among diverse *Neisseria *spp. indicating the occurrence of a horizontal genetic transfer event between *N. meningitidis *and non-pathogenic *Neisseria*. Three features shared between ST-11 meningococci and non-pathogenic *Neisseria *have now been described: (i) the presence of isotype I *tbpB *genes (ii) the identical sequences upstream of the *pilE *gene and (iii) the analogous genetic organisation of the *opcA *negative locus.

A revised nomenclature was proposed according to the published guidelines [31]. The scheme now distinguishes isotype I *tbpB *genes into two new families: *tbpB*_*A *_and *tbpB*_*B *_the former contained *tbpB *genes closely related to ST-11 clonal complex meningococci, the latter included the more distantly related *tbpB *genes belonging to many non-pathogenic *Neisseria *species. The scheme also separates isotype II *tbpB *genes into three new families: *tbpB*_*C *_comprising *tbpB *genes from *N. meningitidis*, *N. lactamica*, *N. polysaccharea *and *N. gonorrhoeae *isolates, *tbpB*_*D *_consisting of *tbpB *genes from *N. lactamica *and *N. meningitidis *isolates and finally, *tbpB*_*E *_containing *N. gonorrhoeae tbpB *genes.

## Methods

### Growth of isolates and DNA preparation

The non-pathogenic *Neisseria *and *N. gonorrhoeae *isolates used in this study are listed in Table 2. Isolates were cultured overnight on GC agar (Difco) supplemented with 1% isovitalex (Oxoid) and grown at 37°C in the presence of 10% CO_2_. Boiled cell suspensions were prepared for each isolate. Briefly, a PBS solution of overnight GC grown bacteria was boiled for 5 minutes, centrifuged and the supernatant stored at +4°C before being directly used for PCR.

### Nucleotide sequence determination

Amplification and sequencing of *tbpB *genes were completed using primers listed in Table 3. Degenerate primers were used for some of the sequencing steps. PCR products were PEG purified and either sequenced directly or cloned using the TOPO PCR TA cloning kit for sequencing (Invitrogen). Nucleotide sequence determination was carried out using the Li-Cor Global IR^2 ^system along with the Sequitherm Excel II DNA sequencing kit (ScienceTec, France). Additional sequencing was carried out by cycle sequencing with BigDye Ready Reaction Mix (Applied Biosystems) according to manufacturer's instructions and using an ABI 377 automated DNA sequencer.

**Table 3:** Primers used in this study

**Primer**	**Primer base sequence (5' – 3')**	**Application**	**Location from 5' end**
OTG6687	CAATCCATTGGTAAATCAG	*tbpB *forward primer	6
OTG6689 [54]	GCCGTCTGAAGCCTTATTC	*tbpB *reverse primer	Intergenic space
seqtbpBI-for1	CTAYAAAGGSARHRAWCCTTCC	Isotype I *tbpB *sequencing	603
seqtbpBI-for2	CCGATTTYGGKMTGACYAG	Isotype I *tbpB *sequencing	817
seqtbpBI-rev1	CCRCCTTCCTGATTGGAGG	Isotype I *tbpB *sequencing	1931
seqtbpBI-rev2	CTGAAATGCCGCCTTATTGCC	Isotype I *tbpB *sequencing	1486
seqtbpBII-for1	GACGGYTATATYTTYTATMAMGG	Isotype II *tbpB *sequencing	585
seqtbpBII-for2	GAAACCAARSAACATCCCTTTG	Isotype II *tbpB *sequencing	1032
seqtbpBII-rev1	GAAGCATTGCCGCTCCAGC	Isotype II *tbpB *sequencing	1901
seqtbpBII-rev2	CTGTTCCGCCGTTTKTACC	Isotype II *tbpB *sequencing	1460

### Data manipulation and analysis

The *tbpB *nucleotide sequences were assembled using the Staden sequence analysis package [38] and all sequences aligned manually in the Seqlab alignment program (Genetics Computer Group, Madison, Wis.). Phylogenetic analysis was undertaken using the software package ClonalFrame version 1.1, which is a statistical model-based method initially described for inferring bacterial clonal relationships using multilocus sequence data [28]. In the present study, over 50,000 iterations were performed with every hundredth tree sampled after which a 95% majority-rule consensus tree was derived. Annotation was then undertaken by importing the tree into the Molecular Evolutionary Genetics Analysis software package (MEGA version 4.0) [29].

The level of sequence diversity between *tbpB *genes was assessed by calculating *p-*distances within each *tbpB *family revealing the proportion (*p*) of nucleotide sites at which sequences differed. These analyses were conducted using MEGA. The number of fixed differences and shared polymorphisms were obtained using the software DnaSP [39]. Old and new accession numbers for *tbpB *genes are listed in table 2.

## Authors' contributions

OBH participated in the planning of this study, performed all experimental work, data analysis and drafted the manuscript. MCJM participated in writing the manuscript. BR participated in the planning of this study, coordinated the study and assisted in writing the manuscript.

## References

[B1] Knapp JS (1988). Historical perspectives and identification of Neisseria and related species. Clin Microbiol Rev.

[B2] Vedros NA, Krieg NR and Holt JG (1984). Genus I. Neisseria Trevissan 1885. Bergey's Manual of systematic bacteriology.

[B3] Gerbase AC, Rowley JT, Mertens TE (1998). Global epidemiology of sexually transmitted diseases. Lancet.

[B4] Rosenstein NE, Perkins BA, Stephens DS, Popovic T, Hughes JM (2001). Meningococcal disease. N Engl J Med.

[B5] Otto BR, Verweij-van Vught AM, MacLaren DM (1992). Transferrins and heme-compounds as iron sources for pathogenic bacteria. Crit Rev Microbiol.

[B6] Prinz T, Meyer M, Pettersson A, Tommassen J (1999). Structural characterization of the lactoferrin receptor from Neisseria meningitidis. J Bacteriol.

[B7] Schryvers AB, Morris LJ (1988). Identification and characterization of the human lactoferrin-binding protein from Neisseria meningitidis. Infect Immun.

[B8] Ala'Aldeen DA, Borriello SP (1996). The meningococcal transferrin-binding proteins 1 and 2 are both surface exposed and generate bactericidal antibodies capable of killing homologous and heterologous strains. Vaccine.

[B9] Schryvers AB, Morris LJ (1988). Identification and characterization of the transferrin receptor from Neisseria meningitidis. Mol Microbiol.

[B10] Lewis LA, Dyer DW (1995). Identification of an iron-regulated outer membrane protein of Neisseria meningitidis involved in the utilization of hemoglobin complexed to haptoglobin. J Bacteriol.

[B11] Lewis LA, Gray E, Wang YP, Roe BA, Dyer DW (1997). Molecular characterization of hpuAB, the haemoglobin-haptoglobin-utilization operon of Neisseria meningitidis. Mol Microbiol.

[B12] Stojiljkovic I, Hwa V, de Saint Martin L, O'Gaora P, Nassif X, Heffron F, So M (1995). The Neisseria meningitidis haemoglobin receptor: its role in iron utilization and virulence. Mol Microbiol.

[B13] Stojiljkovic I, Larson J, Hwa V, Anic S, So M (1996). HmbR outer membrane receptors of pathogenic Neisseria spp.: iron-regulated, hemoglobin-binding proteins with a high level of primary structure conservation. J Bacteriol.

[B14] Rokbi B, Renauld-Mongenie G, Mignon M, Danve B, Poncet D, Chabanel C, Caugant DA, Quentin-Millet MJ (2000). Allelic diversity of the two transferrin binding protein B gene isotypes among a collection of Neisseria meningitidis strains representative of serogroup B disease: implication for the composition of a recombinant TbpB-based vaccine. Infect Immun.

[B15] Rokbi B, Mignon M, Caugant DA, Quentin-Millet MJ (1997). Heterogeneity of tbpB, the transferrin-binding protein B gene, among serogroup B Neisseria meningitidis strains of the ET-5 complex. Clin Diagn Lab Immunol.

[B16] Linz B, Schenker M, Zhu P, Achtman M (2000). Frequent interspecific genetic exchange between commensal Neisseriae and Neisseria meningitidis. Mol Microbiol.

[B17] Zhu P, van der Ende A, Falush D, Brieske N, Morelli G, Linz B, Popovic T, Schuurman IG, Adegbola RA, Zurth K, Gagneux S, Platonov AE, Riou JY, Caugant DA, Nicolas P, Achtman M (2001). Fit genotypes and escape variants of subgroup III Neisseria meningitidis during three pandemics of epidemic meningitis. Proc Natl Acad Sci U S A.

[B18] Bentley SD, Vernikos GS, Snyder LA, Churcher C, Arrowsmith C, Chillingworth T, Cronin A, Davis PH, Holroyd NE, Jagels K, Maddison M, Moule S, Rabbinowitsch E, Sharp S, Unwin L, Whitehead S, Quail MA, Achtman M, Barrell B, Saunders NJ, Parkhill J (2007). Meningococcal genetic variation mechanisms viewed through comparative analysis of serogroup C strain FAM18. PLoS Genet.

[B19] Seiler A, Reinhardt R, Sarkari J, Caugant DA, Achtman M (1996). Allelic polymorphism and site-specific recombination in the opc locus of Neisseria meningitidis. Mol Microbiol.

[B20] Zhu P, Morelli G, Achtman M (1999). The opcA and (psi)opcB regions in Neisseria: genes, pseudogenes, deletions, insertion elements and DNA islands. Mol Microbiol.

[B21] Wang JF, Caugant DA, Morelli G, Koumare B, Achtman M (1993). Antigenic and epidemiologic properties of the ET-37 complex of Neisseria meningitidis. J Infect Dis.

[B22] Aho EL, Urwin R, Batcheller AE, Holmgren AM, Havig K, Kulakoski AM, Vomhof EE, Longfors NS, Erickson CB, Anderson ZK, Dawlaty JM, Mueller JJ (2005). Neisserial pilin genes display extensive interspecies diversity. FEMS Microbiol Lett.

[B23] Zhu P, Klutch MJ, Derrick JP, Prince SM, Tsang RS, Tsai CM (2003). Identification of opcA gene in Neisseria polysaccharea: interspecies diversity of Opc protein family. Gene.

[B24] Claus H, Stoevesandt J, Frosch M, Vogel U (2001). Genetic isolation of meningococci of the electrophoretic type 37 complex. J Bacteriol.

[B25] Cornelissen CN, Anderson JE, Sparling PF (1997). Characterization of the diversity and the transferrin-binding domain of gonococcal transferrin-binding protein 2. Infect Immun.

[B26] Renauld-Mongenie G, Lins L, Krell T, Laffly L, Mignon M, Dupuy M, Delrue RM, Guinet-Morlot F, Brasseur R, Lissolo L (2004). Transferrin-binding protein B of Neisseria meningitidis: sequence-based identification of the transferrin-Binding site confirmed by site-directed mutagenesis. J Bacteriol.

[B27] DeRocco AJ, Cornelissen CN (2007). Identification of transferrin-binding domains in TbpB expressed by Neisseria gonorrhoeae. Infect Immun.

[B28] Didelot X, Falush D (2007). Inference of bacterial microevolution using multilocus sequence data. Genetics.

[B29] Tamura K, Dudley J, Nei M, Kumar S (2007). MEGA4: Molecular Evolutionary Genetics Analysis (MEGA) Software Version 4.0. Mol Biol Evol.

[B30] Rokbi B, Maitre-Wilmotte G, Mazarin V, Fourrichon L, Lissolo L, Quentin-Millet MJ (1995). Variable sequences in a mosaic-like domain of meningococcal tbp2 encode immunoreactive epitopes. FEMS Microbiol Lett.

[B31] Demerec M, Adelberg EA, Clark AJ, Hartman PE (1966). A proposal for a uniform nomenclature in bacterial genetics. Genetics.

[B32] Rokbi B, Mazarin V, Maitre-Wilmotte G, Quentin-Millet MJ (1993). Identification of two major families of transferrin receptors among Neisseria meningitidis strains based on antigenic and genomic features. FEMS Microbiol Lett.

[B33] Irwin SW, Averil N, Cheng CY, Schryvers AB (1993). Preparation and analysis of isogenic mutants in the transferrin receptor protein genes, tbpA and tbpB, from Neisseria meningitidis. Mol Microbiol.

[B34] Renauld-Mongenie G, Poncet D, Mignon M, Fraysse S, Chabanel C, Danve B, Krell T, Quentin-Millet MJ (2004). Role of transferrin receptor from a Neisseria meningitidis tbpB isotype II strain in human transferrin binding and virulence. Infect Immun.

[B35] Anderson JE, Sparling PF, Cornelissen CN (1994). Gonococcal transferrin-binding protein 2 facilitates but is not essential for transferrin utilization. J Bacteriol.

[B36] Gray-Owen SD, Loosmore S, Schryvers AB (1995). Identification and characterization of genes encoding the human transferrin-binding proteins from Haemophilus influenzae. Infect Immun.

[B37] Luke NR, Campagnari AA (1999). Construction and characterization of Moraxella catarrhalis mutants defective in expression of transferrin receptors. Infect Immun.

[B38] Staden R (1996). The Staden sequence analysis package. Mol Biotechnol.

[B39] Rozas J, Sanchez-DelBarrio JC, Messeguer X, Rozas R (2003). DnaSP, DNA polymorphism analyses by the coalescent and other methods. Bioinformatics.

[B40] Perrin A, Bonacorsi S, Carbonnelle E, Talibi D, Dessen P, Nassif X, Tinsley C (2002). Comparative genomics identifies the genetic islands that distinguish Neisseria meningitidis, the agent of cerebrospinal meningitis, from other Neisseria species. Infection and Immunity.

[B41] Perrin A, Nassif X, Tinsley C (1999). Identification of regions of the chromosome of Neisseria meningitidis and Neisseria gonorrhoeae which are specific to the pathogenic Neisseria species. Infection and Immunity.

[B42] Qvarnstrom Y, Swedberg G (2002). Sulphonamide resistant commensal Neisseria with alterations in the dihydropteroate synthase can be isolated from carriers not exposed to sulphonamides. BMC Microbiol.

[B43] Veron M, Thibault P, L. O (1959). [Neisseria mucosa (Diplococcus mucosus Lingelsheim). I. Bacteriological description and study of its pathogenicity]. Ann Inst Pasteur (Paris).

[B44] Zhu P, Klutch MJ, Bash MC, Tsang RS, Ng LK, Tsai CM (2002). Genetic diversity of three lgt loci for biosynthesis of lipooligosaccharide (LOS) in Neisseria species. Microbiology.

[B45] DC S, Arking D, Tong Y (2001). Analysis of lipooligosaccharide biosynthesis in the Neisseriaceae. Journal of Bacteriology.

[B46] Branham SE (1930). A new meningococcus-like organism (Neisseria flavescens m.sp.) from epidemic meningitis.. Public Health Report.

[B47] Taha MK, Marchal C (1990). Conservation of Neisseria gonorrhoeae pilus expression regulatory genes pilA and pilB in the genus Neisseria. Infection and Immunity.

[B48] Barrett SJ, Sneath PHA (1994). A numerical phenotypic taxonomic study of the genus Neisseria. Microbiology.

[B49] Guibourdenche M, Popoff MY, Riou JY (1986). Deoxyribonucleic acid relatedness among Neisseria gonorrhoeae, N. meningitidis, N. lactamica, N. cinerea and "Neisseria polysaccharea". Ann Inst Pasteur Microbiol.

[B50] Spratt BG, Bowler LD, Zhang QY, Zhou J, Smith JM (1992). Role of interspecies transfer of chromosomal genes in the evolution of penicillin resistance in pathogenic and commensal Neisseria species. J Mol Evol.

[B51] Zhu P, Tsang RS, Tsai CM (2003). Nonencapsulated Neisseria meningitidis strain produces amylopectin from sucrose: altering the concept for differentiation between N. meningitidis and N. polysaccharea. J Clin Microbiol.

[B52] Anand CM, Ashton F, Shaw H, Gordon R (1991). Variability in growth of Neisseria polysaccharea on colistin-containing selective media for Neisseria spp. J Clin Microbiol.

[B53] Berger U (1985). First isolation of Neisseria polysacchareae species nova in the Federal Republic of Germany. Eur J Clin Microbiol.

[B54] Legrain M, Rokbi B, Villeval D, Jacobs E (1998). Characterization of genetic exchanges between various highly divergent tbpBs, having occurred in Neisseria meningitidis. Gene.

